# A root-knot nematode effector manipulates the rhizosphere microbiome for establishing parasitism relationship with hosts

**DOI:** 10.3389/fmicb.2023.1217863

**Published:** 2023-07-19

**Authors:** Rui Liu, Mengfei Chen, Boliang Liu, Kaiwei Huang, Zhenchuan Mao, Huixia Li, Jianlong Zhao

**Affiliations:** ^1^College of Plant Protection, Gansu Agricultural University/Biocontrol Engineering Laboratory of Crop Diseases and Pests of Gansu Province, Lanzhou, Gansu, China; ^2^State Key Laboratory of Vegetable Biobreeding, Institute of Vegetables and Flowers, Chinese Academy of Agricultural Sciences, Beijing, China; ^3^School of Life Sciences, Sun Yat-Sen University, Guangzhou, China

**Keywords:** rhizosphere microbiome, *Meloidogyne incognita*, *Arabidopsis thaliana*, MiMIF-2 effector protein, parasitism

## Abstract

**Introduction:**

Root-knot nematode (RKN; *Meloidogyne* spp.) is one of the most infamous soilborne plant diseases, causing severe crop losses every year. Effector proteins secreted by RKNs play crucial roles during plant-nematode interaction. However, less is known about whether RKN effector proteins can impact the rhizosphere microbial environment.

**Methods:**

In this study, we investigated the rhizosphere microbiome community of *MiMIF-2* (a plant immunity-modulating effector) transgenic *Arabidopsis thaliana* with or without nematode infection using the Illumina high-throughput sequencing analysis.

**Results and discussion:**

The results showed that the bacterial species richness index increased, while the fungi species richness index decreased in *M. incognita*-infected *MiMIF-2* transgenic *A. thaliana* plants. The relative abundance of genera such as *Clitopilus, Komagataeibacter, Lactobacillus, Prevotella, Moritella, Vibrio, Escherichia-Shigella*, and *Pseudomonas* was reduced in *MiMIF-2* transgenic *A. thaliana* plants compared to wild type, but was significantly increased after inoculation with *M. incognita*. The Cluster of Orthologous Genes (COG) function classification analysis revealed a decrease in the relative abundance of defense mechanisms, secondary metabolite biosynthesis, transport, and nematode infection catabolism-related functions in *MiMIF-2* lines compared to the wild type. These differences may be the reason for the increased susceptibility of *MiMIF-2* transgenic *A. thaliana* to nematode infection. Our results provide a new insight into RKN effector proteins and their association with the microbial community, host, and plant pathogens, which will lead to the exploration of new innovative ideas for future biological control of RKNs.

## 1. Introduction

Root-knot nematodes (RKNs), belonging to the genus *Meloidogyne*, are among the most destructive plant-parasite nematodes (Trudgill, 1997). RKNs have an exceptionally large host range, with more than 5,500 plant hosts, and cause economic losses worth more than 100 billion dollars every year (Abad et al., 2008; Elling, 2013). Among RKNs, *Meloidogyne incognita* is an obligate, biotrophic pathogen of many plant species and is believed to be the most harmful nematode species in traditional as well as protected agriculture (Jones et al., 2013). RKNs build remarkably sophisticated interactions with their host plants (Vieira and Gleason, 2019). During the interaction, the motile second-stage juveniles (J2s) of *M. incognita* enter the host root tip and migrate intercellularly to reach the host vascular cells to form hypertrophied feeding cells called giant cells (GCs) (Favery et al., 2016). In recent decades, most studies focus on elucidating the molecular mechanism of plant-nematode interactions. However, less is known about the functions of the rhizosphere microbiome during RKN parasitism.

It is widely recognized that the extent of microbial colonization of plants is a crucial factor affecting their health (Berendsen et al., 2012). There are intricate interactions in plants, microbes, and nematodes (Bais et al., 2006). In 2012, studies revealed the core endophytic bacterial microbiome in the model plant *Arabidopsis thaliana*, where the dominant phyla are *Proteobacteria, Bacteroidetes*, and *Firmicutes* (Bulgarelli et al., 2012; Lundberg et al., 2012). Tens of thousands of plant-associated bacterial strains have been isolated, and they modulate plant growth and development, thereby increasing the yield of crops (Hardoim et al., 2008). Any disruption to this “plant-soil-microorganism” interaction can play a significant role in the occurrence of diseases (Classen et al., 2015). The rhizosphere microbiome has different mechanisms to effectively control RKNs. Certain bacteria, such as *Bacillus cereus* (Yin et al., 2021), *Pseudomonas aeruginosa* (Siddiqui and Shaukat, 2003), and *Brevundimonas diminuta* (Zheng et al., 2008), can suppress nematode infection. Conversely, other bacteria such as *Chitinophaga* (Baquiran et al., 2013) and *Pedobacter* (Tian et al., 2011) can promote the growth of nematodes. Many fungi have also been reported to inhibit RKNs. *Verticillium, Paecilomyces lilacinus* (Wang et al., 2010), and *Pochonia chlamydosporia* (Galeano et al., 2003) are known to inhibit RKNs parasitism. By investigating the microbial communities affected by RKNs in tomato roots and analyzing their functional characteristics in the interactions between microbes, plants, and nematodes, studies have made an intriguing finding: the pathogenicity of nematodes led to a decrease in the prevalence of the prominent endophytic groups, specifically *Streptomyces* and *Pseudomonas* (Tian et al., 2015).

Effectors are small molecular compounds secreted by nematodes into host tissues to alter host physiology and assist in the infection process (Hogenhout et al., 2009). In the last 20 years, a large number of effectors of *M. incognita* have been reported, which are involved in promoting infection, migration, and parasitism of nematodes in plant roots (Mejias et al., 2019, 2022; Truong et al., 2021; Zhao et al., 2021). We previously reported that *M. incognita* secretes macrophage migration inhibitory factor-like proteins (MiMIFs) that suppress plant immunity and facilitate nematode parasitism. *MiMIF-2* exhibited enzyme activities and has played a potential role in plant salicylic acid synthesis (Zhao et al., 2019, 2020). Despite the essential roles in modulating plant immune responses of nematode effectors, their relationship with rhizosphere microorganisms and hosts is largely unknown.

A previous report has shown that plant pathogens secrete effectors to manipulate the host microbiome (Snelders et al., 2018). Recently, a report also showed that the soil-borne fungal plant pathogen *Verticillium dahlia* secreted the effector Vdave1, which manipulated the host microbiome to facilitate colonization in tomato and cotton (Snelders et al., 2020). Despite several metagenomic studies conducted to explore the diversity of root microbial community upon RKN infection (Wu et al., 2022; Lu et al., 2023), the key factors that affect RKN development and the potential mechanisms of RKN inhibition have not been fully explored. In this study, we focused on the rhizosphere microbiome of *MiMIF-2* ectopic expression in *A. thaliana*, with or without inoculation of *M. incognita*. The results showed that the effector-driven effect on the species of fungi and bacteria contributes to the plasticity of the host rhizosphere microbiome, which is critical for *M. incognita* parasitism.

## 2. Materials and methods

### 2.1. Plant growth conditions and nematode culture

*MiMIF-2* transgenic *A. thaliana* lines were previously described (Zhao et al., 2019). Wild-type (WT) *A. thaliana* (Col-0) was used as a control. Surface-sterilized *A. thaliana* seeds were placed on Murashige and Skoog Medium (MS) (Sigma, St. Louis, MO, USA) and were incubated at 22°C with a 12-h photoperiod. Seedlings of 15 days old plants were transplanted into soil and were grown in the greenhouse at 22°C and 65% relative humidity with a 16-h light/8-h dark photoperiod.

Tomato plants (*Solanum lycopersicum* var. “Baiguo”) were used to reproduce *M. incognita* nematode culture. Infective pre-parasitic second-stage juveniles (pre-J2s) were collected after egg hatching (Zhao et al., 2019).

### 2.2. Sample collection of rhizosphere soil

Homozygous T3 *MiMIF-2* transgenic *A. thaliana* lines and WT were challenged with 200 pre-parasitic J2s of *M. incognita*, indicated by MiMIF-2+N and WT+N. WT and *MiMIF-2* transgenic *A. thaliana* lines of uninoculated nematodes were used as the controls, indicated by WT and MiMIF-2. All the plants were grown in the laboratory of the Institute of Vegetable and Flower Research of the Chinese Academy of Agricultural Sciences, Beijing, China (116.19.32°E, 39.57.44°N). Rhizosphere soil samples around the *A. thaliana* roots were collected at 30 days post-inoculation (dpi) with nematodes. After excavating the roots of 30 *Arabidopsis* plants from the soil, the soil that was loosely attached to the roots was carefully removed. The soil that adhered to the *Arabidopsis* root system was brushed loose using a delicate brush. This soil was collected in sterile self-sealing bags and mixed thoroughly, and the amalgamated soil was divided into five equal portions. Each treatment was replicated five times with five seedlings per replication (Lundberg et al., 2012).

### 2.3. DNA extraction and PCR amplification

Microbial community genomic DNA was extracted using 0.5 g of soil per sample employing the E.Z.N.A.^®^ soil DNA Kit (Omega Bio-tek, Norcross, GA, USA) as per the manufacturer's recommendations. The quality of the DNA extract was confirmed on 1% agarose gel, whereas the DNA concentration and purity were determined using the NanoDrop 2000 UV-vis spectrophotometer (Thermo Scientific, Wilmington, USA). Amplification of the hypervariable region, V3–V4 of the bacterial 16S rRNA gene, was performed using the primer pair 338F (5'-ACTCCTACGGGAGGCAGCAG-3') and 806R (5'-GGACTACHVGGGTWTCTAAT-3'), whereas the primer set SSU0817 forward (5′-TTAGCATGGAATAATRRAATAGGA-3′) and 1196 reverse (5′-TCT GGACCTGGTGAGTTTCC-3′) was used to amplify the V5–V7 region of the 18S rDNA gene through the ABI GeneAmp^®^ 9700 PCR thermocycler (ABI, CA, USA).

The PCR method used for the amplification comprised of an initial denaturation at 95°C for 3 min, followed by 27 cycles of denaturation at 95°C for 30 s, annealing at 55°C for 30 s, and extension at 72°C for 45 s in each cycle with a final extension at 72°C for 10 min after which the reaction was maintained at 4°C. The PCR mixtures contained 5× TransStart FastPfu buffer 4 μl, 2.5 mM dNTPs 2 μl, forward primer (5 μM) 0.8 μl, reverse primer (5 μM) 0.8 μl, TransStart FastPfu DNA polymerase 0.4 μl, template DNA 10 ng, and ddH_2_O to make a final volume of 20 μl. PCR amplifications were performed in triplicate. The PCR products were electrophoresed using 2% agarose gel, and the band of interest was eluted using the AxyPrep DNA Gel Extraction Kit (Axygen Biosciences, Union City, CA, USA) as per the recommendations of the manufacturer, and the eluted DNA was quantified using the Quantus™ Fluorometer (Promega, USA).

### 2.4. Illumina MiSeq sequencing

Purified amplicons pooled in equimolar volumes were paired-end sequenced (2 × 300) on an Illumina MiSeq platform (Illumina, San Diego, USA) following the standard protocols recommended by Majorbio Bio-Pharm Technology Co. Ltd. (Shanghai, China). The raw reads were deposited into the NCBI Sequence Read Archive (SRA) database (accession number: PRJNA954129).

### 2.5. Bioinformatics and statistical analyses

Splicing and filtering were performed on raw data to secure valid data and to ensure the accuracy and reliability of the results. Operational taxonomic units (OTUs) were clustered using 97% similarity. The Chao and Shannon indices were computed to figure out the alpha diversity and to determine the species richness in each soil sample. A principal coordinates analysis (PCoA) of each sample formulated on the Bray–Curtis distances was accomplished using the R package vegan (version 2.1). Linear discriminant analysis (LDA) effect size measurements (LEfSe) was performed to search for significantly different (*P* < 0.05) taxa between the two groups. Significantly different taxa were used to generate taxonomic cladograms, which illustrated the differences between sample classes on the website http://huttenhower.sph.harvard.edu/galaxy.

Graphical representations were created using GraphPad Prism 5 (GraphPad Software, Inc., La Jolla, CA, USA). The mean and standard error for each group of data were calculated using the ANOVA and Tukey's honestly significant difference test (*P* < 0.05) using SPSS 19.0 (SPSS Inc., Chicago, IL, USA).

## 3. Results

### 3.1. Sequencing and metagenome assembly

After Illumina paired-end sequence, quality evaluation, data filtering, and integration, a total of 933,427 bacterial 16S rRNA sequences (Supplementary Table S1) and 1,169,253 effective fungal 18S rDNA sequences (Supplementary Table S2) were generated from 20 samples. A total of 2,299 bacterial OTUs were assigned to 38 different phyla: WT contained 28, MiMIF-2 contained 32, WT+N contained 30, and MiMIF-2+N contained 32 (Table 1). A total of 492 fungal OTUs were assigned to 9 different phyla: WT contained 9, MiMIF-2 contained 9, WT+N contained 9, and MiMIF-2+N contained 8 (Table 2).

**Table 1:** General features of the high-throughput bacteria sequencing results in different samples.

**Sample**	**Number of sequences**	**Number of OTU**	**Number of genera**	**Number of family**	**Number of order**	**Number of class**	**Number of phylum**
WT	47,072	1,542	518	292	179	80	28
MiMIF-2	47,606	1,649	521	302	183	78	32
WT+N	44,768	1,532	517	295	188	81	30
MiMIF-2+N	47,240	1,615	522	300	187	81	32

**Table 2:** General features of the high-throughput fungal sequencing results in different samples.

**Sample**	**Number of sequences**	**Number of OTU**	**Number of genera**	**Number of family**	**Number of orders**	**Number of classes**	**Number of phyla**
WT	45,016	264	86	65	43	24	9
MiMIF-2	49,222	348	100	76	49	27	9
WT+N	43,558	262	82	62	41	23	9
MiMIF-2+N	50,644	328	95	74	49	26	8

### 3.2. Composition and diversity of the rhizosphere microbiome with or without nematode infection

**Figure 1:**
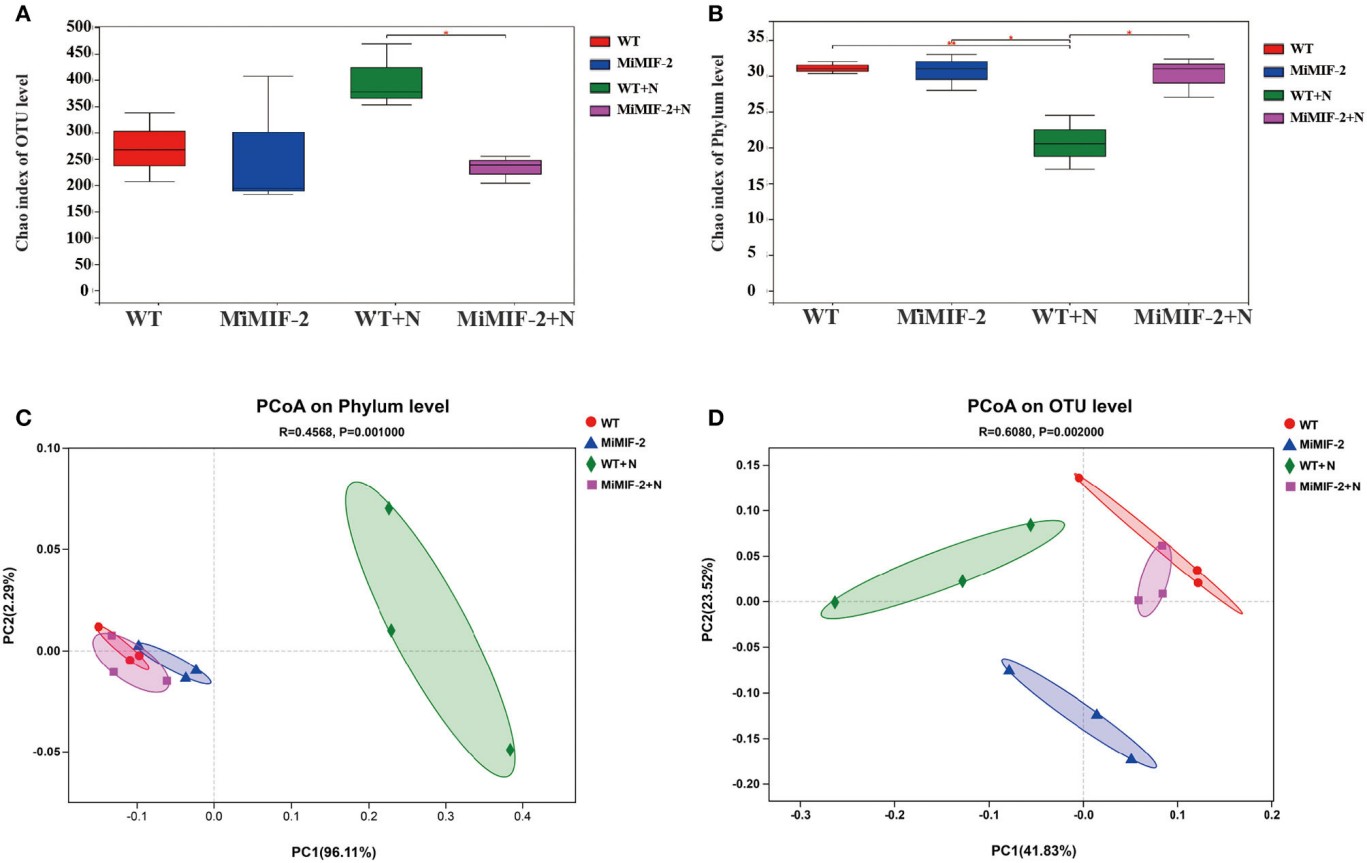
Diversity analysis: Alpha-diversity of the *Arabidopsis* rhizo-compartment microbiota in different samples. **(A)** fungal species richness (Chao1) index of OUT level; **(B)** bacterial species richness (Chao) index of phylum level (^*^indicates *P* < 0.05; ^**^indicates *P* < 0.01; Wilcoxon rank sum test). PCoA of Bray–Curtis distances reveals that soil type is a major source of bacterial **(C)** and fungal **(D)** community variation in both the rhizosphere and roots.

By comparing the α-diversities of the fungal species richness (Chao1) index, there was a significant difference between the before and after inoculation nematodes. However, the fungal species richness in *MiMIF-2* was significantly reduced compared to WT, when inoculated with *M. incognita* (*P* < 0.05, Wilcoxon rank sum test; Figure 1A).

In the case of bacterial species richness index, however, *MiMIF-2* lines showed a significant increase compared to WT after inoculation with *M. incognita*. The bacterial species richness (Chao1) index was also reduced in wild-type *Arabidopsis* when compared with uninoculated WT (Figure 1B). Interestingly, the species richness index of WT inoculated with *M. incognita* was significantly lower than that of uninoculated samples (Figure 1B). The increase in bacterial species diversity might be the reason for the increased nematode susceptibility in *MiMIF-2*.

Statistical analysis of Bray–Curtis distances (β-diversity) was involved in the comparison of the composition of microbial communities. It was performed to determine the community treatment relationship between the samples. Concerning the phylum level, PCoA was performed on each sample, and the distribution of WT+N and other samples was significantly different for bacteria (Figure 1C). At the OTU level, PCoA of Bray–Curtis distances (β-diversity) revealed that WT, MiMIF-2, WT+N, and MiMIF-2+N microbiota in four types of soil exhibited a clear separation (Figure 1D).

**Figure 2:**
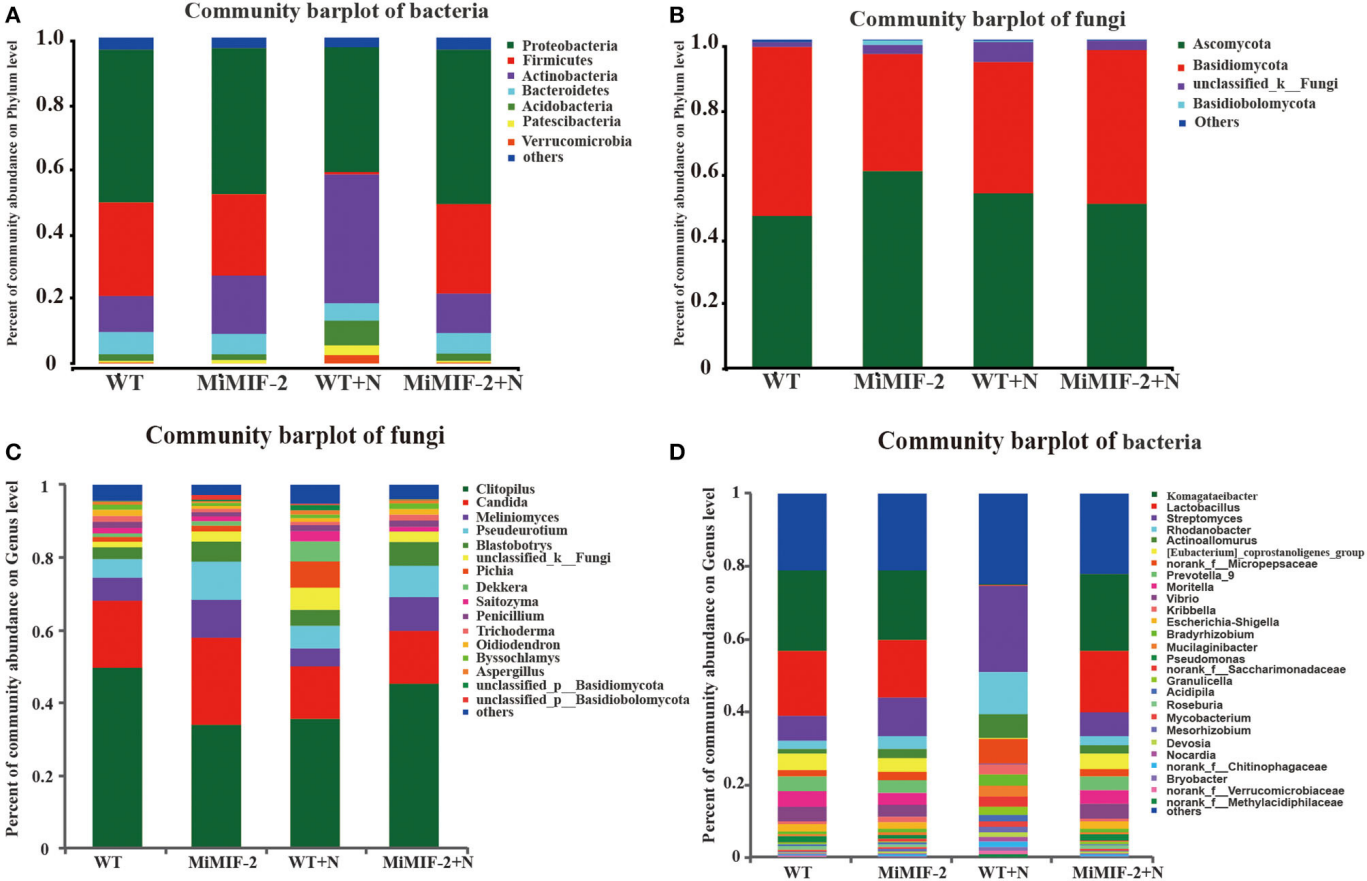
Community composition at the phylum and genus level of the rhizosphere soil samples of wild-type *Arabidopsis thaliana* (WT), *MiMIF-2* transgenic *A. thaliana* lines (*MiMIF-2*), inoculated *M. incognita MiMIF-2* transgenic *A. thaliana* lines (MiMIF-2+N) and WT (WT+N). Bacterial **(A)** and fungal **(B)** community composition at the phylum level. Fungal **(C)** and bacterial **(D)** community composition at the genus level.

OTUs of the four samples were assigned corresponding taxonomies based on the combined search results against the Greengenes and NCBI databases. The relative abundance of different phyla in the four samples was analyzed, of which *Proteobacteria, Actinobacteria*, and *Firmicutes* were the dominant bacterial phyla (Figure 2A). *Ascomycota* and *Basidiomycota* were the dominant fungal phyla in all soil samples, accounting for more than 90% of the total abundance in each sample (Figure 2B). After inoculation with nematodes, *Firmicutes* of WT+N were significantly reduced compared to uninoculated samples (Figure 2A). In community composition, *Clitopilus, Candida, Meliniomyces*, and *Pseudeurotium* were the dominant fungal genera in all soil samples (Figure 2C) whereas in the bacterium community composition, *Komagataeibacter, Lactobacillus, Streptomyce*s, and *Rhodanobacter* were the dominant bacterial genera in all the rhizosphere bacteria. *Lactobacillus* of WT+N were significantly reduced compared to uninoculated samples. On the contrary, *Streptomyces, Rhodanobacter*, and *Actinoallomurus* of WT+N species increased compared with other samples (Figure 2D).

### 3.3. Predominant microbe species affected by *M. incognita*

**Figure 3:**
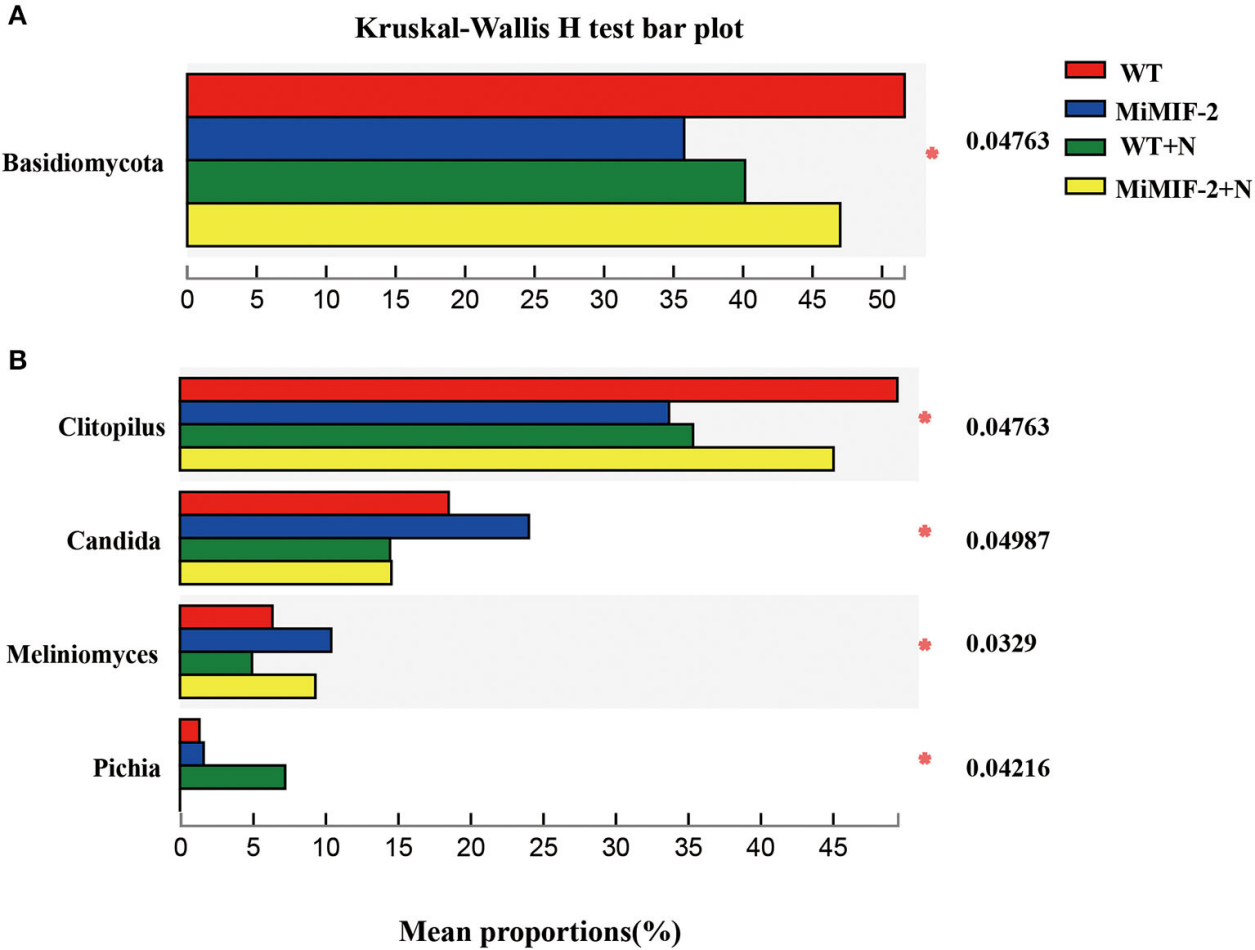
**(A, B)** The different fungal relative abundances of major phylum and genera among the different compartments. WT (red), MiMIF-2 (blue), WT+N (green), and MiMIF-2+N (yellow). The Kruskal–Wallis *H*-test was used to evaluate the significance of the difference between the indicated groups (*n* = 3, *indicates *P* < 0.05).

The relative abundances of major fungal phyla and genus, including *Basidiomycota, Clitopilus*, and *Candida*, were different in *MiMIF-2* transgenic *A. thaliana* (Figure 3A). Phylum *Basidiomycota* abundance in *MiMIF-2* transgenic *A. thaliana* decreased compared with WT; however, the relative abundances increased after inoculation with *M. incognita*. Similar results were obtained during the analysis at the genus level. *Clitopilus* behaved similarly as *Basidiomycota*. *Candida* was abundant in *MiMIF-2* transgenic *A. thaliana*; however, no differences were observed after inoculation with *M. incognita* (Figure 3B).

**Figure 4:**
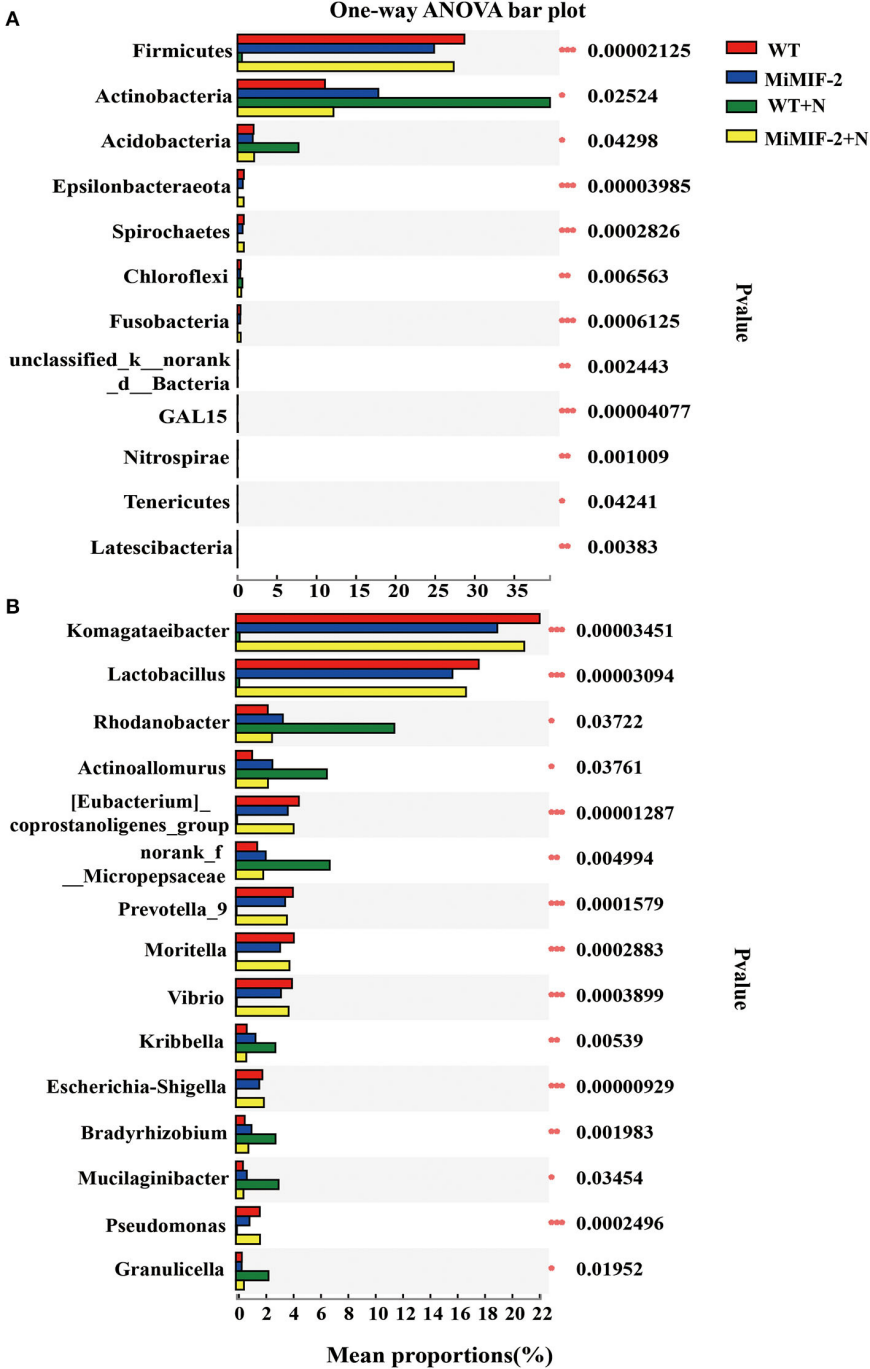
The different bacterial relative abundances of major phylum **(A)** and genera **(B)** among the different compartments. WT (red), MiMIF-2 (blue), WT+N (green), and MiMIF-2 + N (yellow). The Kruskal–Wallis *H*-test was used to evaluate the significance of the difference between the indicated groups (*n* = 3, ^*^indicates *P* < 0.05; ^**^indicates *P* < 0.01; ^***^indicates *P* < 0.001).

The analysis of bacterial phylum-level relative abundances revealed significant differences among *Firmicutes, Actinobacteria, Acidobacteria, Epsilonbacteraeota, Spirochetes*, and *Chloroflexi*. *Firmicutes, Epsilonbacteraeota, Spirochetes*, and *Fusobacteria* were significantly higher in MiMIF-2+N compared to those in WT+N. However, the richness of the phylum of WT was significantly reduced after inoculation with nematodes. *Actinobacteria, Acidobacteria*, and *Chloroflexi* were reduced in MiMIF-2+N compared to WT+N (Figure 4A). A similar phenomenon occurs at the genus-level relative abundances. More than 15 genera showed significant differences. For instance, *Komagataeibacter, Lactobacillus, Prevotella, Moritella, Vibrio, Escherichia-Shigella*, and *Pseudomonas* were reduced in *MiMIF-2* lines compared to those in WT. However, these genera were significantly higher in MiMIF-2+N compared to those in WT+N (Figure 4B).

### 3.4. Microbial communities with statistically significant differences

**Figure 5:**
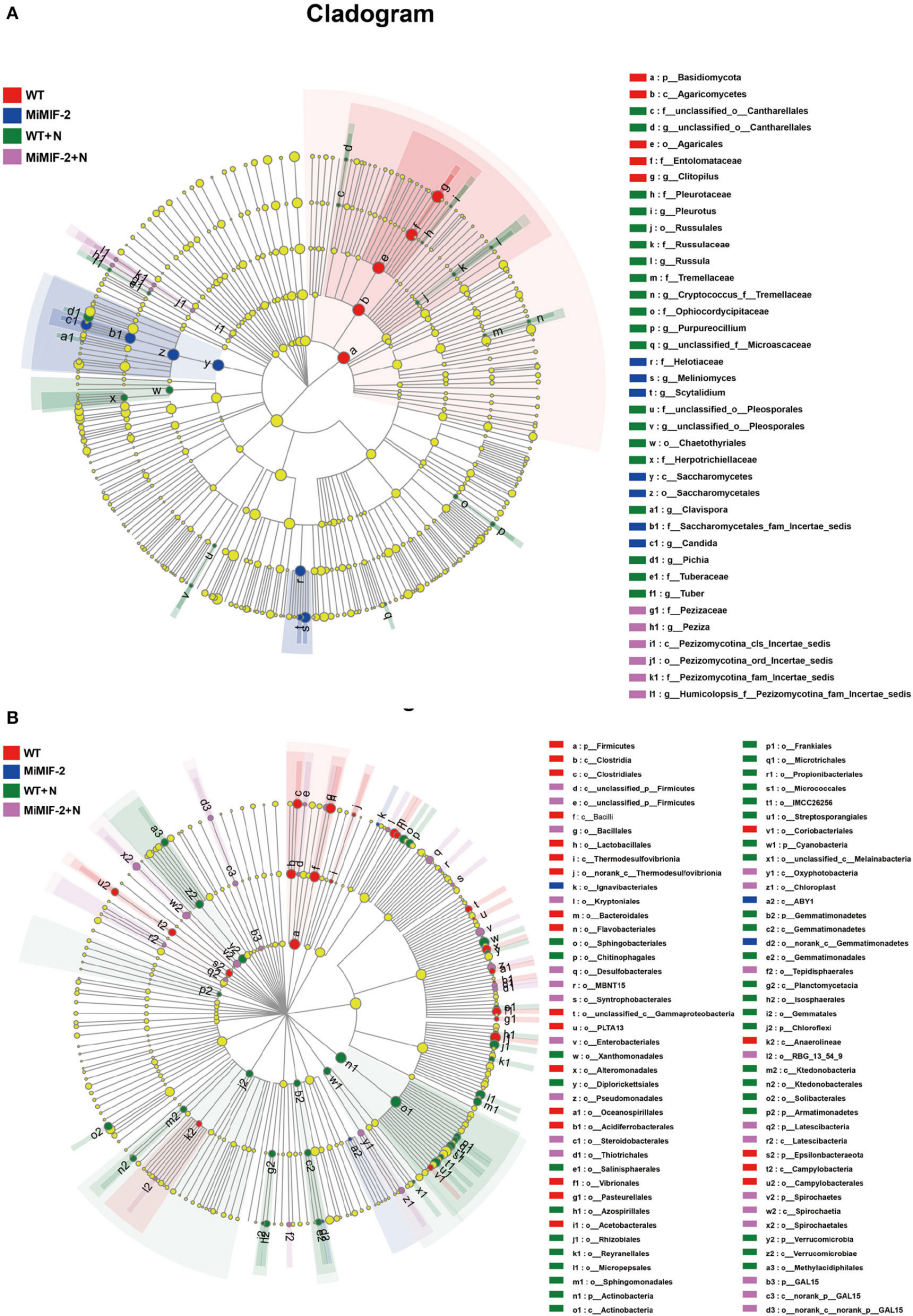
LEfSe cladogram of the aggregated groups of WT, MiMIF-2, WT+N, and MiMIF-2+N. A range of bacterium **(A)** and fungus **(B)** taxa from phylum to genus level was associated with WT (red), MiMIF-2 (blue), WT+N (green), and MiMIF-2+N (violet) (α = 0.05, LDA > 4.0, the size of circles is proportional to each taxon's mean relative abundance). The yellow circles represent the absence of significantly different taxa.

The LEfSe was used to identify the dominant phylotypes responsible for the differences among the samples. The groups were shown in cladograms, and LDA scores of 4 (Supplementary Tables S3, S4) or greater were confirmed using LEfSe (Figure 5). In MiMIF-2+N, 11 groups of bacteria were significantly enriched, while no fungi were detected at a significant level. The bacteria with the highest LDA value in MiMIF-2+N was *Firmicutes* (logarithmic LDA score = 4.73). In WT+N, 4 groups of bacteria and 3 groups of fungi were significantly enriched. The bacterium and fungus with the highest LDA value in WT+N were *Gammaproteobacteria* (logarithmic LDA score = 4.57) and *Pleurotaceae* (logarithmic LDA score = 4.87), respectively. In WT, 5 groups of bacteria and 5 groups of fungi were detected at a significant level. The bacterium and fungus with the highest LDA value in WT were *Proteobacteria* (logarithmic LDA score = 4.98) and *Entolomataceae* (logarithmic LDA score = 4.96), respectively. In MiMIF-2, 4 groups of bacteria and 6 groups of fungi were detected at a significant level. The bacterium and fungus with the highest LDA value in MiMIF-2 were *Actinobacteriota* (logarithmic LDA score = 4.79) and *Saccharomycetes* (logarithmic LDA score = 4.84), respectively. Community structure and predominant taxa of different samples may account for differences in sensitivity to nematodes.

### 3.5. Functional annotations and functional guilds for the different rhizosphere microbiomes

**Figure 6:**
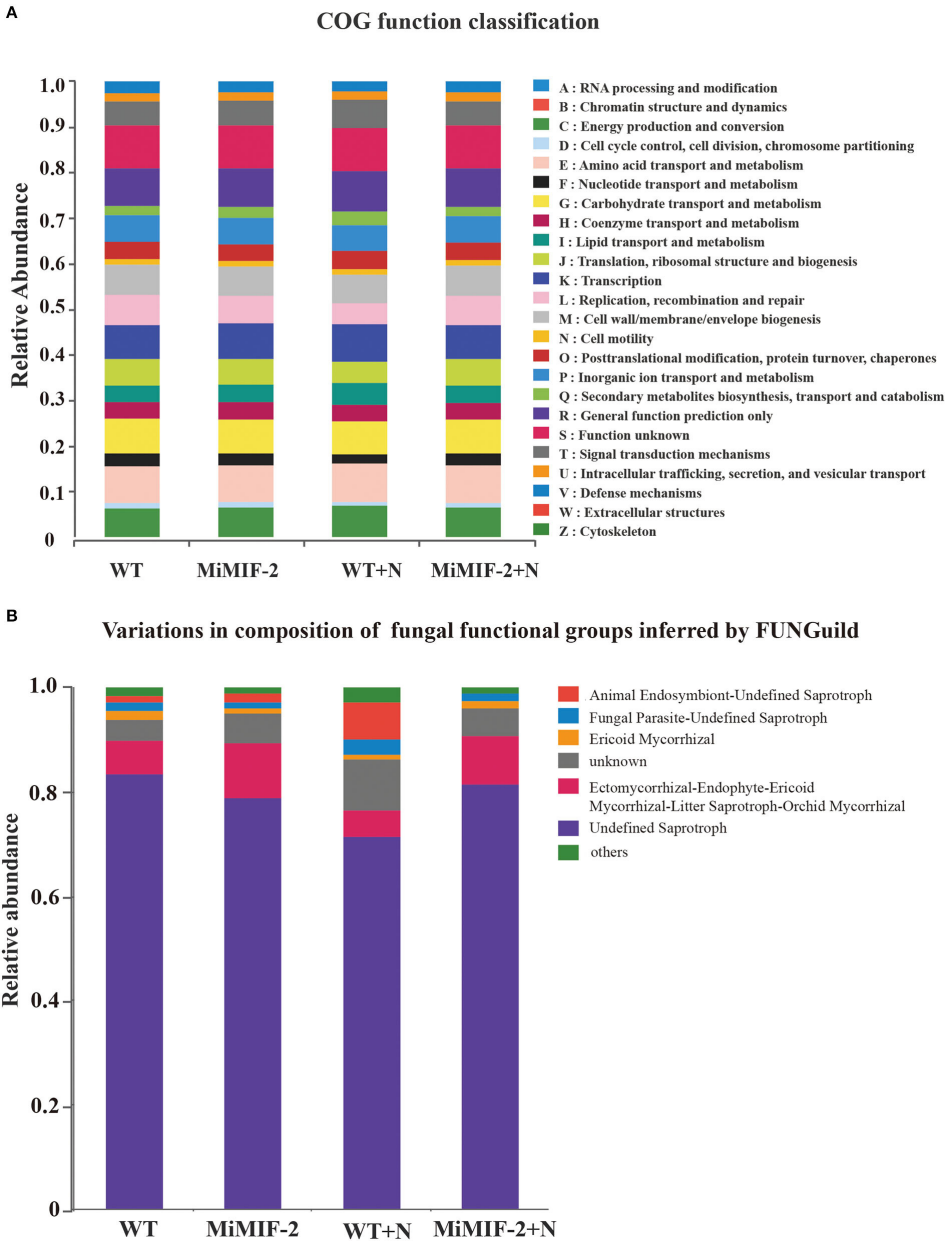
Relative abundance of the PICRUSt inferred function in different samples **(A)**. Relative abundance of variations in the composition of fungal functional groups inferred by FUNGuild **(B)**. The relative abundance is calculated by averaging the abundances of duplicate samples.

The relative abundance of PICRUSt inferred function is shown in Figure 6A. All soil rhizosphere microorganisms consist mainly of energy production and conversion, amino acid transport and metabolism, carbohydrate transport and metabolism, cell wall/membrane/envelope biogenesis, and general function prediction. Whether or not the nematode is inoculated, the relative abundance of all the COG function classifications in WT was slightly larger than that in *MiMIF-2* lines (Supplementary Table S5). For example, defense mechanisms, secondary metabolites biosynthesis, transport, and catabolism may be related to nematode infection.

We used a manually curated set of designations based on FunGuild, a recently established fungal classification tool with rigorously defined and referenced trophic group assignments. The relative abundance of animal endosymbiont-undefined saprotroph was higher after inoculation with nematodes; however, it was almost none after transferring to the MiMIF-2 effector (Figure 6B).

## 4. Discussion

The rhizosphere microbes associated with plant roots are enormous, in the order of tens of thousands of species, where complex biological and ecological processes occur (Berendsen et al., 2012). It is universally accepted that the host's rhizosphere microbes play a significant role in its health and that hosts actively shape their microbiomes to prevent or suppress disease development (Pieterse et al., 2001; Lugtenberg and Kamilova, 2009). In previous studies, rhizosphere microbes play a key role in the relationship among the plant, soil, and pathogens (Trivedi et al., 2020). It has been well established that pathogens secrete effector molecules of various kinds during attempted host ingress to promote disease development, many of which target essential components of the host immune system. Our previous study showed that *MiMIF-2* was an effector protein secreted from the cuticle of *M. incognita*, and it enters host cells during nematode parasitism; MiMIF-2 effector could suppress plant immunity by interacting with plant annexin proteins (Zhao et al., 2019). We provided evidence that salicylic acid (SA)-related marker genes and the content of SA were significantly reduced when *MiMIF-2* transgenic *Arabidopsis* was challenged against pathogens (Zhao et al., 2020), roles of *MiMIF-2* and annexins in root responses to soil microbes are expected. During the plant-microbiome interaction, beneficial microorganisms will improve plant resistance to pathogens and stresses. However, pathogens, such as RKNs, have evolved sophisticated means to interfere with plant immunities and recruit microbiome suits for themselves (Trivedi et al., 2020). Thus, we speculate that *MiMIF-2* expression in planta will alter host immune responses and also manipulate the microbiome in favor of nematode parasitism. In recent years, similar reports have shown other pathogens manipulating the host immune system by secreting effectors to promote disease development, especially those of filamentous pathogens (Rovenich et al., 2014; Snelders et al., 2018). Moreover, researchers demonstrated that a fungal plant pathogen uses effector proteins to modulate microbiome compositions inside and outside the host (Snelders et al., 2020).

Community analysis of tomato root-associated with healthy and nematode-infected tomatoes indicated that nematode pathogenesis led to a decrease in the abundance of the main endophytic bacteria *Streptomycetaceae* and *Pseudomonadales* (Tian et al., 2015). After *M. incognita* infected tomato, the main components of the root microorganisms were *Proteobacteria, Actinobacteria, Bacteroidetes*, and *Firmicutes* (Cao et al., 2015). Rhizosphere microbiome structures difference alters to tomato wilt resistance (Kwak et al., 2018). There was a significant relationship between RKN disease and rhizosphere microbial diversity (Lu et al., 2023). It has been reported that many *Actinomyces* have strong biocontrol potential in the egg mass and adult worms of nematodes (Sun et al., 2006). *Streptomyces* are important producers of antibiotics and toxic metabolites, and *Streptomyces* species have been found to control fungal pathogens and nematodes, which are considered promising biological control agents (Krechel et al., 2002). The analysis of rhizosphere microorganisms' diversity revealed that the presence of *MiMIF-2* caused an increase in the species richness of *Actinobacteria, Acidobacteria, Firmicutes*, and *Proteobacteria*, ultimately leading to a promoted sensitivity to nematodes.

Pathogenic microorganisms can manipulate the host microbiome through the immune system of salicylic acid (Wu et al., 2022), reactive oxygen species (Song et al., 2021), PTI immune response (Topalovi et al., 2020), etc., which is conducive to parasitism. In this study, the relative abundance of the rhizosphere microorganisms in *MiMIF-2* transgenic lines decreased compared with the wild type in secondary metabolism synthesis and defense response. Collectively, we speculate that the MiMIF-2 effector plays a role in manipulating the rhizosphere microbial to promote the parasitism of *M. incognita* through immune response changes of the host.

## 5. Conclusion

Taken together, our study showed that *MiMIF-2* expression in plants has an effect on the aggregation of bacteria and fungi. Interestingly, there was an increase in bacterial species and a decrease in fungi species when *MiMIF-2* transgenic *A. thaliana* plants were infected with RKNs. We observed a decrease in defense mechanisms and secondary metabolites in *MiMIF-2* transgenic *A. thaliana*, which could explain their increased susceptibility to nematode infection. These findings shed new light on the role of plant-parasitic nematode effector proteins and their interaction with microbial communities, plants, and pathogens, providing new clues for the biological control of RKNs.

## Data availability statement

The datasets presented in this study can be found in online repositories. The names of the repository/repositories and accession number(s) can be found at: https://www.ncbi.nlm.nih.gov/genbank/, PRJNA954129.

## Author contributions

RL, MC, and KH: performed the experiments. RL, BL, ZM, JZ, and HL: contributed to the data analysis, discussion, wrote, reviewed, and corrected the manuscript. All authors contributed to the article and approved the submitted version.
