# Supplementary material for: A root-knot nematode effector manipulates the rhizosphere microbiome for establishing parasitism relationship with hosts
Source: Front Microbiol. 2023 Jul 19;14:1217863. doi: 10.3389/fmicb.2023.1217863 (PMC10395084; doi:10.3389/fmicb.2023.1217863)
Supplement: Supplementary file 1 [file Table_1.DOCX]

**Supplemental Tables**

**Table S1:** The bacteria sequence number of different samples.

**Table S2:** The fungi sequence number of different samples.

**Table S3:** Bacteria LDA discriminant result table of LEfSe cladogram of the aggregated groups.

**Table S4:** Fungal LDA discriminant result table of LEfSe cladogram of the aggregated groups.

**Table S5:** COG functional classification statistics.

**Table S1 The bacteria sequence number of different samples**

| **Sample** | **Seq_num** | **Average length** | **Total bases** |
| --- | --- | --- | --- |
| WT_1 | 63627 | 415.095384 | 26411274 |
| WT_2 | 44445 | 413.6196648 | 18383326 |
| WT_3 | 42204 | 415.2157615 | 17523766 |
| WT_4 | 35663 | 415.1531559 | 14805607 |
| WT_5 | 49421 | 414.9594707 | 20507712 |
| MiMIF-2_1 | 46751 | 416.1219225 | 19454116 |
| MiMIF-2_2 | 56893 | 415.3672508 | 23631489 |
| MiMIF-2_3 | 46324 | 414.8836 | 19219069 |
| MiMIF-2_4 | 50122 | 415.961 | 20848797 |
| MiMIF-2_5 | 37940 | 415.5104 | 15764466 |
| WT+N_1 | 53834 | 414.8583 | 22333480 |
| WT+N_2 | 46891 | 414.3029 | 19427079 |
| WT+N_3 | 48887 | 415.597 | 20317290 |
| WT+N_4 | 36629 | 415.532 | 15220523 |
| WT+N_5 | 37597 | 414.2126 | 15573151 |
| MiMIF-2+N_1 | 43868 | 415.6037 | 18231701 |
| MiMIF-2+N_2 | 40742 | 415.908 | 16944923 |
| MiMIF-2+N_3 | 50524 | 416.0359 | 21019800 |
| MiMIF-2+N_4 | 57975 | 416.017 | 24118588 |
| MiMIF-2+N_5 | 43090 | 416.0109 | 17925909 |

**Table S2 The fungi sequence number of** **different samples**

| **Sample** | **Seq_num** | **Average length** | **Total bases** |
| --- | --- | --- | --- |
| WT_1 | 43308 | 293.606493 | 12715510 |
| WT_2 | 40606 | 291.7523026 | 11846894 |
| WT_3 | 47981 | 291.9280965 | 14007002 |
| WT_4 | 46964 | 295.4753002 | 13876702 |
| WT_5 | 46219 | 287.6772972 | 13296157 |
| MiMIF-2_1 | 56808 | 272.7057105 | 15491866 |
| MiMIF-2_2 | 44622 | 295.3415132 | 13178729 |
| MiMIF-2_3 | 49586 | 279.0331344 | 13836137 |
| MiMIF-2_4 | 44334 | 284.6821401 | 12621098 |
| MiMIF-2_5 | 50759 | 290.9730097 | 14769499 |
| WT+N_1 | 41716 | 295.7971042 | 12339472 |
| WT+N_2 | 39399 | 293.6387979 | 11569075 |
| WT+N_3 | 52108 | 294.2602671 | 15333314 |
| WT+N_4 | 42732 | 292.684686 | 12507002 |
| WT+N_5 | 41834 | 297.7461156 | 12455911 |
| MiMIF-2+N_1 | 47346 | 290.5175516 | 13754844 |
| MiMIF-2+N_2 | 51541 | 267.5399585 | 13789277 |
| MiMIF-2+N_3 | 43140 | 307.0046824 | 13244182 |
| MiMIF-2+N_4 | 56971 | 262.1018764 | 14932206 |
| MiMIF-2+N_5 | 54224 | 275.0765713 | 14915752 |

**Table S3 Bacteria LDA discriminant result table of LEfSe cladogram of the aggregated groups**

| **Species name** | **group** | **Mean** | **LDA_value** | **Pvalue** |
| --- | --- | --- | --- | --- |
| p__Actinobacteriota.c__Actinobacteria | MiMIF-2+N | 5.381898 | 4.649452 | 0.006841 |
| p__Firmicutes.c__Bacilli.o__Paenibacillales | MiMIF-2+N | 4.682223 | 4.190564 | 0.001229 |
| p__Proteobacteria | WT | 5.676797 | 4.983385 | 0.003538 |
| p__Actinobacteriota | MiMIF-2 | 5.487788 | 4.789469 | 0.003104 |
| p__Actinobacteriota.c__Thermoleophilia.o__Solirubrobacterales.f__67_14 | MiMIF-2 | 4.372549 | 4.039245 | 0.002421 |
| p__Actinobacteriota.c__Thermoleophilia | MiMIF-2 | 4.643603 | 4.048401 | 0.002687 |
| p__Proteobacteria.c__Alphaproteobacteria.o__Micropepsales | WT | 4.517757 | 4.009433 | 0.002978 |
| p__Actinobacteriota.c__Actinobacteria.o__Streptomycetales.f__Streptomycetaceae | MiMIF-2+N | 5.212203 | 4.691945 | 0.003068 |
| p__Actinobacteriota.c__Thermoleophilia.o__Solirubrobacterales.f__67_14.g__norank_f__67_14 | MiMIF-2 | 4.372549 | 4.040962 | 0.002421 |
| p__Proteobacteria.c__Alphaproteobacteria.o__Rhizobiales.f__Rhizobiaceae | WT+N | 4.855918 | 4.324583 | 0.003515 |
| p__Firmicutes.c__Bacilli.o__Bacillales.f__Bacillaceae.g__Bacillus | MMIF-2+N | 5.009616 | 4.526457 | 0.001444 |
| p__Proteobacteria.c__Alphaproteobacteria | WT | 5.485477 | 4.776509 | 0.00357 |
| p__Actinobacteriota.c__Actinobacteria.o__Streptomycetales.f__Streptomycetaceae.g__Streptomyces | MiMIF-2+N | 5.212203 | 4.691945 | 0.003068 |
| p__Proteobacteria.c__Gammaproteobacteria.o__Burkholderiales | WT | 4.984841 | 4.468704 | 0.003325 |
| p__Proteobacteria.c__Gammaproteobacteria.o__Burkholderiales.f__Comamonadaceae | WT+N | 4.492862 | 4.052642 | 0.001717 |
| p__Firmicutes.c__Bacilli.o__Bacillales.f__Bacillaceae | MiMIF-2+N | 5.014406 | 4.528773 | 0.001444 |
| p__Proteobacteria.c__Alphaproteobacteria.o__Rhizobiales | WT | 5.229378 | 4.532585 | 0.002632 |
| p__Proteobacteria.c__Alphaproteobacteria.o__Rhizobiales.f__Rhizobiaceae.g__Allorhizobium_Neorhizobium_Pararhizobium_Rhizobium | WT+N | 4.776837 | 4.237952 | 0.003392 |
| p__Proteobacteria.c__Gammaproteobacteria | WT+N | 5.238204 | 4.57576 | 0.00307 |
| p__Firmicutes.c__Bacilli.o__Bacillales | MiMIF-2+N | 5.019942 | 4.534675 | 0.001444 |
| p__Firmicutes | MiMIF-2+N | 5.288828 | 4.739444 | 0.001579 |
| p__Firmicutes.c__Bacilli.o__Paenibacillales.f__Paenibacillaceae | MiMI-2+N | 4.682223 | 4.190569 | 0.001229 |
| p__Firmicutes.c__Bacilli | MiMIF-2+N | 5.218909 | 4.662784 | 0.001368 |
| p__Actinobacteriota.c__Actinobacteria.o__Streptomycetales | MiMIF-2+N | 5.212203 | 4.691945 | 0.003068 |
| p__Proteobacteria.c__Gammaproteobacteria.o__Burkholderiales.f__Oxalobacteraceae | WT | 4.471108 | 4.050809 | 0.003236 |

**Table S4 Fungal LDA discriminant result table of LEfSe cladogram of the aggregated groups**

| Species name | group | Mean | LDA_value | Pvalue |
| --- | --- | --- | --- | --- |
| p__Ascomycota.c__Leotiomycetes.o__  Helotiales.f__Helotiaceae | MiMIF-2 | 5.023209 | 4.487097 | 0.044448 |
| p__Ascomycota.c__Leotiomycetes.o__  Helotiales.f__Helotiaceae.g__Meliniomyces | MiMIF-2 | 5.018408 | 4.457403 | 0.032902 |
| p__Ascomycota.c__Saccharomycetes.o__  Saccharomycetales | MiMIF-2 | 5.511996 | 4.718213 | 0.023744 |
| p__Ascomycota.c__Saccharomycetes.o__  Saccharomycetales.f__Saccharomycetales_  fam_Incertae_sedis | MiMIF-2 | 5.381409 | 4.707511 | 0.049871 |
| p__Basidiomycota.c__Agaricomycetes.o__Agaricales | WT | 5.694451 | 4.915189 | 0.047629 |
| p__Basidiomycota | WT | 5.712816 | 4.900735 | 0.047629 |
| p__Basidiomycota.c__Agaricomycetes.o__Agaricales.f__Entolomataceae | WT | 5.694375 | 4.955715 | 0.047629 |
| p__Ascomycota.c__Saccharomycetes.o__  Saccharomycetales.f__Saccharomycetales_  fam_Incertae_sedis.g__Candida | MiMIF-2 | 5.381251 | 4.665175 | 0.049871 |
| p__Ascomycota.c__Saccharomycetes | MiMIF-2 | 5.511996 | 4.838963 | 0.023744 |
| p__Basidiomycota.c__Agaricomycetes.o__Agaricales.f__Pleurotaceae | WT+N | 1.417653 | 4.878636 | 0.013249 |
| p__Basidiomycota.c__Agaricomycetes.o__Agaricales.f__Entolomataceae.g__Clitopilus | WT | 5.694375 | 4.899703 | 0.047629 |
| p__Basidiomycota.c__Agaricomycetes.o__Agaricales.f__Pleurotaceae.g__Pleurotus | WT+N | 1.417653 | 4.072162 | 0.013249 |
| p__Basidiomycota.c__Agaricomycetes | WT | 5.695101 | 4.910119 | 0.047629 |
| p__Ascomycota.c__Saccharomycetes.o__Saccharomycetales.f__Pichiaceae.g__Pichia | WT+N | 4.86008 | 4.539354 | 0.042164 |
|  |  |  |  |  |

**Table S5 COG functional classification statistics**

| **Category** | | **MiMIF-2+N** | **WT** | **WT+N** | **MiMIF-2** | | **Description** |
| --- | --- | --- | --- | --- | --- | --- | --- |
| A | 15073 | | 15632 | 16722 | 15467 | | RNA processing and modification |
| B | 13162 | | 17020 | 17396 | 13379 | | Chromatin structure and dynamics |
| C | 1946147 | | 2210590 | 2290770 | 2004820 | | Energy production and conversion |
| D | 242771 | | 285290 | 296542 | 251459 | | Cell cycle control, cell division, chromosome partitioning |
| E | 2455756 | | 2905795 | 2992208 | 2506396 | | Amino acid transport and metabolism |
| F | 607000 | | 698006 | 725240 | 627548 | | Nucleotide transport and metabolism |
| G | 1932369 | | 2077878 | 2165248 | 1966350 | | Carbohydrate transport and metabolism |
| H | 1032786 | | 1171087 | 1211207 | 1074215 | | Coenzyme transport and metabolism |
| I | | 1384357 | 1554799 | 1624656 | 1424717 | | Lipid transport and metabolism |
| J | | 1256862 | 1484661 | 1540741 | 1307136 | | Translation, ribosomal structure and biogenesis |
| K | | 2274583 | 2444160 | 2553793 | 2315957 | | Transcription |
| L | | 1272451 | 1536104 | 1586457 | 1325006 | | Replication, recombination and repair |
| M | | 1598271 | 1950405 | 2019619 | 1672015 | | Cell wall/membrane/envelope biogenesis |
| N | | 343274 | 476459 | 479441 | 353755 | | Cell motility |
| O | | 1035645 | 1263821 | 1311143 | 1084423 | | Posttranslational modification, protein turnover, chaperones |
| P | | 1644319 | 2024130 | 2088680 | 1678524 | Inorganic ion transport and metabolism | |
| Q | | 890362 | 949354 | 989250 | 906921 | Secondary metabolites biosynthesis, transport and catabolism | |
| R | | 2424815 | 2744238 | 2845071 | 2492710 | General function prediction only | |
| S | | 2604855 | 3199494 | 3310971 | 2680285 | Function unknown | |
| T | | 1835300 | 2045815 | 2145962 | 1928714 | Signal transduction mechanisms | |
| U | | 453099 | 615320 | 637730 | 478972 | Intracellular trafficking, secretion, and vesicular transport | |
| V | | 503352 | 515452 | 543871 | 523844 | Defense mechanisms | |
| W | | 56 | 175 | 200 | 49 | Extracellular structures | |
| Z | | 9238 | 9333 | 9691 | 9603 | Cytoskeleton | |
